# Whole Grain Consumption and Inflammatory Markers: A Systematic Literature Review of Randomized Control Trials

**DOI:** 10.3390/nu14020374

**Published:** 2022-01-16

**Authors:** Genevieve Milesi, Anna Rangan, Sara Grafenauer

**Affiliations:** 1 Nutrition and Dietetics Group, Sydney Nursing School, Faculty of Medicine and Health, Charles Perkins Centre, The University of Sydney, Camperdown, NSW 2006, Australia; gmil2280@uni.sydney.edu.au (G.M.); anna.rangan@sydney.edu.au (A.R.); 2 Grains & Legumes Nutrition Council, Mount Street, North Sydney, NSW 2060, Australia; 3 School of Medicine & Health, University of New South Wales, Randwick, NSW 2052, Australia

**Keywords:** whole grain, refined grain, inflammation, inflammatory markers, C-reactive protein, tumor necrosis factor, interlukin-6

## Abstract

Whole grain foods are rich in nutrients, dietary fibre, a range of antioxidants, and phytochemicals, and may have potential to act in an anti-inflammatory manner, which could help impact chronic disease risk. This systematic literature review aimed to examine the specific effects of whole grains on selected inflammatory markers from human clinical trials in adults. As per the Preferred Reporting Items for Systematic Reviews (PRISMA) protocol, the online databases MEDLINE, Embase, Cochrane, CINAHL, and Scopus were searched from inception through to 31 August 2021. Randomized control trials (RCTs) ≥ 4 weeks in duration, reporting ≥1 of the following: C-reactive protein (CRP), interleukin-6 (IL-6), and tumor necrosis factor (TNF), were included. A total of 31 RCTs were included, of which 16 studies recruited overweight/obese individuals, 12 had pre-existing conditions, two were in a healthy population, and one study included participants with prostate cancer. Of these 31 RCTs, three included studies with two intervention arms. A total of 32 individual studies measured CRP (10/32 were significant), 18 individual studies measured IL-6 (2/18 were significant), and 13 individual studies measured TNF (5/13 were significant). Most often, the overweight/obese population and those with pre-existing conditions showed significant reductions in inflammatory markers, mainly CRP (34% of studies). Overall, consumption of whole grain foods had a significant effect in reducing at least one inflammatory marker as demonstrated in 12/31 RCTs.

## 1. Introduction

Whole grains are defined by Food Standards Australia and New Zealand (FSANZ), to be ‘… intact, dehulled, ground, cracked or flaked grains where the components–endosperm, germ and bran are present in substantially the same proportions as they exist in the intact grain’ and includes wholemeal [1]. More recently, a consensus definition of whole grain as a food and as an ingredient was published with the aim of assisting in nutrition education and food labeling, but this also provides useful guidance for research [2]. Foods containing whole grains are both higher in nutrients and dietary fiber, as compared to refined grain alternatives, and in observational studies, diets higher in whole grains positively impact chronic disease, such as type 2 diabetes mellitus [3], cardiovascular disease (CVD) [4], certain cancers [4] including colorectal cancer [5,6,7,8], and other influencing risk factors, such as weight [9], and markers for CVD, such as triglyceride and cholesterol levels [10]. In addition, the nutrient bundle within whole grains contains potential anti-inflammatory properties, which is of importance as elevated levels of inflammatory biomarkers are linked to an increase in chronic disease risk [2,3]. The benefits of whole grain foods, including pseudo grains, quinoa, buckwheat, and amaranth, have been known for several decades, and included in the Australian Dietary Guidelines since 1979 [11]. Chronic disease was responsible for 9 out of 10 deaths in Australia in 2018, and 61% of the total burden of disease in Australians in 2017 [12], indicating the potential importance of improved dietary guidance and dietary patterns. However consumption of whole grain foods continues to remain at a low level, with Australian adults only consuming 21 g/day, less than half of the 48 g daily target intake (DTI) [11,13]. Furthermore, diets low in whole grains have been identified as the second greatest dietary risk factor for mortality in the Global Burden of Disease studies [14], highlighting the importance of dietary patterns. 

The anti-inflammatory effects of whole grains can be examined via inflammatory markers, such as C-reactive protein, (CRP), interleukin-6, (IL-6), and tumor necrosis factors (TNF), and can potentially downregulate an inflammatory response [15]. Inflammatory markers change in response to a cascade of internal metabolic processes, where chronic inflammation can lead to chronic disease [15]. 

There is a growing body of evidence linking whole grain consumption with overall health benefits; however, the specific influence of whole grains on inflammatory markers is conflicting [11,16]. To date, systematic reviews of randomized controlled trials (RCTs) have focused on the consumption of whole grains and their association with individual chronic health diseases, such as CVD or T2D [17]. Others have focused specifically on dietary fiber levels in whole grains and associated effects; however, there is no current summation of the literature focusing solely on the consumption of whole grains and their direct effect on inflammatory markers. Although there are two previously published systematic reviews in this area [17,18], an update was necessary that focused only on adults, with a strict criteria for whole grain to meet the accepted definition and to clarify other discrepancies. This systematic literature review aimed to examine the specific effects of whole grains on inflammatory markers from human clinical trials in adults. The intent was to investigate whether the consumption of whole or pseudo grains, over refined grains, resulted in changes in inflammatory markers, based on results in human subjects in studies ≥ 4 weeks duration. 

## 2. Materials and Methods

This systematic literature review of RCT was performed to assess the effect of whole grain consumption on inflammatory markers following the Preferred Reporting Items for Systematic Reviews and Meta-Analysis (PRISMA) guidelines. This study was registered with the International Prospective Register of Systematic Reviews (CRD: pending). 

### 2.1. Eligibility and Exclusion Criteria

The research question ‘Is there an effect of whole grain consumption on measures of inflammation?’ was developed using the Population, Intervention, Comparator, Outcome (PICO) format (Figure S1). Publications needed to meet the following inclusion criteria: (a) RCT, parallel, or cross-over design; (b) studies conducted on humans aged ≥18 years; (c) studies ≥ 4 weeks in duration; (d) studies with interventions including both whole grain and pseudo grain diets, where whole grains included: cereal grains; wheat; including spelt, emmer, einkorn, Khorasan or kamut, durum, and faro; oats, corn/maize, rice, teff, canary seeds, Job’s Tears, barley, sorghum, rye, millet and triticale, and pseudo-cereal grains; amaranth, buckwheat, quinoa, and wild rice; (d) reporting ≥1 of the following serum inflammatory markers: interleukin-6, (IL-6), C-reactive protein, (CRP), tumor necrosis factor (TNF). Full search terms can be found in Table S2. 

The following exclusion criteria were applied; (a) studies conducted on humans < 18 years; (b) study intervention arms not randomized; (c) studies < 4 weeks in duration. Although inflammatory markers were examined by both Jenkins et al. [19] and Kristensen et al. [20], the intervention diet included several foods, not just whole grain foods; therefore, these studies were excluded from the current review.

### 2.2. Search Strategy

The following online databases were searched: Medline, Embase, Cochrane Central Register of Controlled Trials (CENTRAL). Available online: https://ovidsp.ovid.com/ (accessed on 13 December 2021), and CINAHL. Available online: https://www.ebsco.com/ (accessed on 13 December 2021), from database inception until 31 August 2021. In addition, reference lists of eligible studies were scanned and PubMed. Available online: https://pubmed.ncbi.nlm.nih.gov/ (accessed on 13 December 2021).

Was searched manually for any additional studies. The search strategy was designed in Medline and translated for other databases (Table S2). Grey literature, abandoned trials, and any journals published in languages other than English were excluded from the search strategy. 

### 2.3. Study Selection, Data Extraction, and Quality Assessment 

Reviewer G.M extracted all citations into EndNote X9, with duplicates removed manually. Reviewer G.M independently double screened all titles and abstracts, with any uncertainty and assistance from S.G. Following title and abstract screening, a full-text screen was completed on the remaining articles by two independent reviewers (S.G. and G.M.). Reviewers met and resolved any discrepancies, with any remaining uncertainty resolved by a third reviewer (A.R.). 

A data extraction form was created in a Microsoft^®^ Excel^®^ spreadsheet (Microsoft 365 MSO Version 2109.14430.20306 Redmond, WA, USA) to facilitate the retrieval and storage of relevant data. Extracted data included study design (parallel or cross-over), study duration, participant characteristics, number of participants, control and intervention diet, outcomes measured, and results obtained (baseline, endpoint data, and *p*-value). 

The included studies were reviewed for risk of bias using the Cochrane Risk of Bias tool (Rob2) for RCTs [21]. Reviewer G.M assessed studies to determine if each study had low, some concerns, or high risk of bias. Assessment criteria included risk of bias arising from recruitment of subjects, the randomization process, deviations from the interventions, missing data, measurement of outcome, or selection of the reported result. A second reviewer (S.G.) was consulted over any uncertainties. 

### 2.4. Data Analysis

Tabulation of studies including reported mean ± SD of baseline and endpoint data and statistical significance (*p*-value) for within-group and between-group intervention changes for each study, and for studies with multiple intervention arms was performed. Within the included studies, outcomes were considered statistically significant when *p* < 0.05. The outcome measures were maintained as per the study units due to the differences in the various experimental methods used. Studies were then categorized into population groups based on the authors’ description of participants: healthy individuals, overweight or obese individuals, individuals with pre-existing conditions, and others (prostate cancer). 

## 3. Results

### 3.1. Search Results and Study Selection

**Figure 1:**
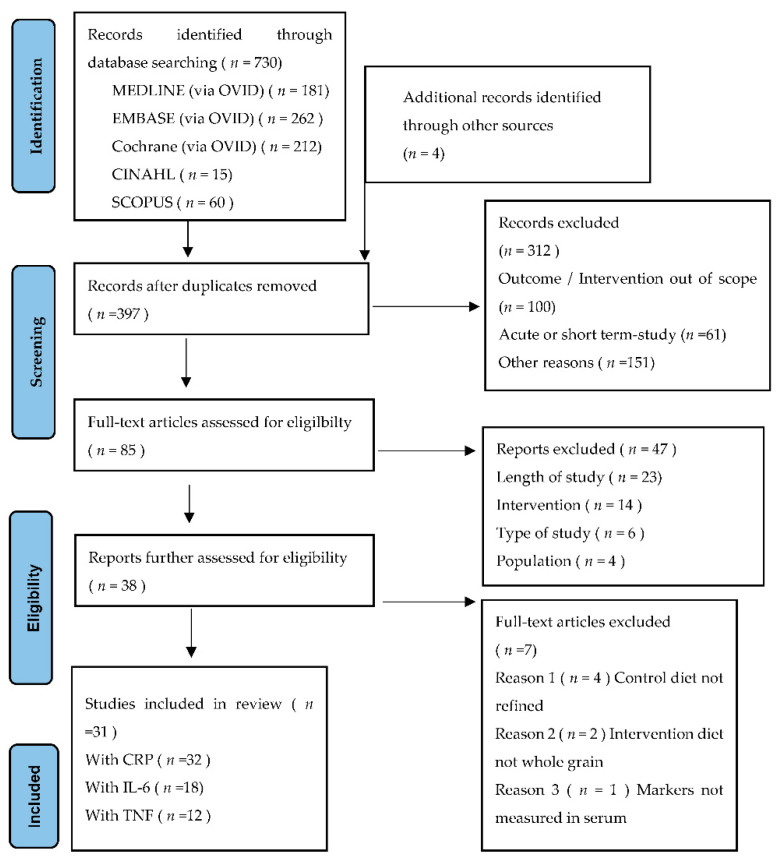
Preferred Reporting Items for Systematic Reviews and Meta-Analysis (PRISMA) flow diagram for study selection.

The initial search, conducted on 31 August 2021, returned a total of 730 studies. An additional four studies were identified from the reference list of eligible studies and manual searches from PubMed. After the removal of duplicates, 397 were screened for the title and abstract, with a further 312 studies excluded. A full-text review was completed on the remaining 85 records, with 47 removed due to the type of study, study did not have an adult population, or length of the RCT < 4 weeks. The remaining 38 studies were further assessed, with six removed as the control or intervention diet was not whole or refined grains and one measured inflammation in fecal matter, not from blood serum. A remaining total of 31 RCTs met the inclusion criteria and were included in the systematic review (Figure 1). 

### 3.2. Study Characteristics

Of the 31 studies included in analysis, 16 were parallel RCTs and 15 were crossover trials. Of these studies, three RCTs included two intervention arms, and thus were split into a further three studies [22,23]. Table 1 displays the study characteristics. Two studies comprised whole grain interventions in healthy populations, 16 studies overweight or obese, 12 pre-existing conditions, and one reviewing another disease state: prostate cancer. The studies had a total of 2047 participants, with a mean age of 49.7 (range 20–80 years old) and the mean duration of the study was 12.5 weeks (range 4–24 weeks). 

### 3.3. Risk of Bias

**Figure 2:**
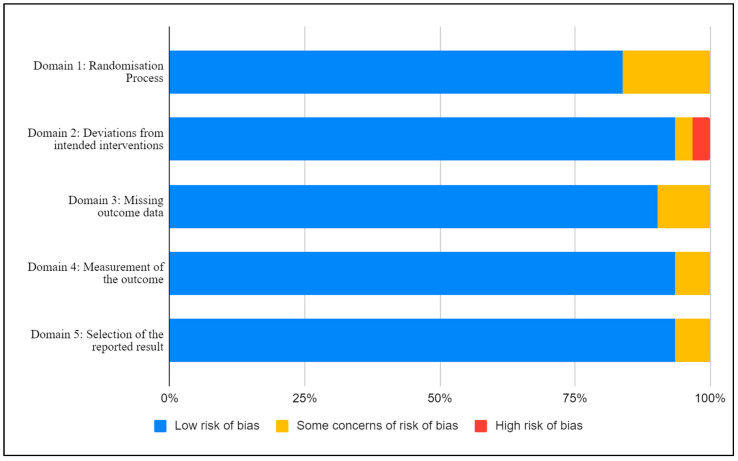
Risk of bias assessment using the revised Cochrane risk-of-bias (RoB 2).

A summary of the within-study risk of bias is shown in Figure 2. The included studies were assessed against the predetermined criteria of the Cochrane RoB2 tool for randomized control and crossover trials [21]. Within Domain 1: Randomization Process, there were five studies with some concerns of bias [24,28,30,31,43], with the remaining studies (*n* = 26) with a low risk of bias. In Domain 2: Deviations from intended intervention, there was one study with a high risk of bias [22], one with some concern [31], and the remainder with a low risk of bias (*n* = 29). Three studies had some risk of bias for Domain 3: Missing outcome data, [28,41,45], and the remainder had a low risk of bias (*n* = 28). Two studies had some risk of bias for both Domain 4: Measurement of the outcome [25,47] and Domain 5: Selection of the reported result [26,31], with the remainder having a low risk of bias (*n* = 29). 

### 3.4. Effect of the Intervention on the Outcome 

#### 3.4.1. Healthy Individuals

Two studies measured the effect of whole grain consumption on healthy individuals, who had a BMI < 25 and with no pre-existing conditions [23,40]. Within these studies, two measured CRP, while only one measured IL-6 and TNF. No marker for the studies looking at healthy individuals showed any level of statistical significance. The details are displayed in Table 2.

#### 3.4.2. Overweight or Obese Individuals

Among the 16 studies in the overweight and obese populations (BMI 25–35), two had two intervention arms [22,47], resulting in 18 studies within this category (Table 3). All 18 studies measured CRP levels, with six of these (33%) observing a statistically significant reduction in CRP levels following whole grain consumption [29,30,32,33,38,40]. Nine of the studies measured IL-6 levels, with one observing a statistically significant change in IL-6 levels after consumption of whole grain foods [40]. A further two of the five total studies measuring TNF also observed a statistically significant change in inflammatory marker levels [32,49]. 

#### 3.4.3. Individuals with Pre-Existing Conditions 

In the 12 studies that reviewed individuals with pre-existing conditions, which included type 2 diabetes [23,35,43,45,50], metabolic syndrome [27,28,31,48], type 2 diabetes and metabolic syndrome [23], acute coronary syndrome [50], and hypercholesterolaemia [42], one study had two intervention arms included in this SLR [23] (Table 4). Of the 11 studies measuring CRP, four observed a statistically significant change [23,31,42,43]. Seven studies measured IL-6 levels, with only one showing a significant change [42]. These seven studies also reviewed TNF levels, with three observing an increase in the level of change between the intervention and the control group, which was statistically significant [28,42,51].

#### 3.4.4. Individuals with Other Conditions

One study had a population that fit outside of the other population groups: males with prostate cancer [52] (Table 5). This study measured CRP and IL-6 levels and whilst the data was not prepared in accordance with other measures, the study observed no statistical level of significance for either. 

## 4. Discussion

Consumption of whole grains in preference to refined grains is known to have improved health benefits, with the broad range of benefits often attributed solely to the presence of dietary fiber [10,53]; however, other components, phytochemicals, fatty acids, amino acids, vitamins, and minerals are all likely to play a role. This review of 31 RCTs found that consumption of whole grain foods had a moderate effect on reducing inflammatory markers, with five of the possible 15 crossover studies [33,38,40,42,50], and seven of 16 parallel studies demonstrating statistically significant changes [23,29,30,31,32,43,49]. Within the population groups studied, the reduction in markers was most often observed in obese and overweight populations, and among those with pre-existing conditions, compared with studies of healthy populations, although there were only two studies in this category. 

Previous systematic reviews and meta analyses, performed by Rahmani et al. [17] and Hajihashemi et al. [18] utilising publications up until 2019, found little evidence of a relationship between whole grain consumption and inflammatory markers. The current review included a total of 13 papers not included in the aforementioned reviews [17,18], six of which were published outside the timeframe utilized by the previous authors [30,31,41,43,44,52], and a further seven were included in the current review due to a variation in the search strategy [23,29,37,39,42,45,51]. 

While the findings of the current study provide some indication that whole grain consumption leads to a downregulation of inflammation, the wide variety of foods classed as whole grain included in the intervention diets varied between studies, from commercially available whole grain products to a specific dose allocated via food items provided by the research group. Of the 31 studies reviewed, 27 provided the intervention foods; however, the remaining four studies [25,32,36,43] only provided guidelines or instructions of which foods to purchase, adding a significant burden for study participants in sourcing and selecting the correct food types, which is a known issue for consumers [54]. Blind compliance checks are problematic and alkylresorcinol levels were only utilized by Harris Jackson et al. [28]; however, this test is only relevant for whole grain wheat and rye [55,56]. Despite this limitation, such biomarkers have been suggested in research to help support dietary assessment of consumption [56]. 

Only three of the 31 studies noted that subjects were instructed to maintain weight for the duration of the study [27,29,42], and only one study controlled for weight in their analysis [50], with all others showing a slight decrease in weight or no data mentioned. In addition, only eight studies recorded or mentioned physical activity or exercise, with six asked to maintain [23,25,27,30,33,41], one asked to record any exercise [32], and one asked to refrain completely [29]. A change in weight either through diet or exercise could be a possible confounder, as it becomes difficult to isolate the changes in inflammatory status as a result of the consumption of whole grain or as a result of the weight (fat) loss [57]. Despite the focus of papers based on the overweight and obese population, only 16 of 30 RCTs measured body fat mass [22,24,25,27,30,31,34,35,37,38,40,41,45,46,48,49], with no consistency in the method or type of body fat measured between studies, making comparisons between studies difficult. Furthermore, the more favorable results within studies of overweight populations are likely due to higher inflammatory marker levels at baseline in comparison to healthy populations. This finding is of particular importance as dietary interventions that result in a reduction in inflammation are important due to the link with reduced risk of chronic diseases [58].

As inflammation is known to increase with age [59] and the average age of the participants was 50 years (20–80 years), future studies could look at potential differences in age groups, or alternatively study a larger population sample segmenting by age, health status, or gender. This would enable the identification of population groups where the diet prescription may be most efficacious. 

Chronic disease remains one of the largest cost contributors to the global burden of disease, with overweight contributing 8.7% of the annual cost of the total burden of disease in Australia in 2019 [12]. On a population level, swapping from refined grains to whole grains has the possibility of reducing the risk of chronic disease, in turn lowering the costs related to the burden of disease. A recent nutrition economics analysis found that a swap to whole grain from refined grain foods could provide significant healthcare cost savings for cardiovascular disease, type 2 diabetes, and cancer, particularly colorectal cancer, for the Australian population [60,61]. 

Further studies investigating the relationship between consumption of whole grain foods over comparable refined grain products and the influence on inflammatory markers are needed to confirm the presence and strength of the relationship. Studies with standardized diets where the single focus of the dietary intervention was whole grain foods compared with refined grain foods would help to narrow the possibility that the intervention diet was responsible for the change in the inflammatory response. Previous research has emphasized the need to accurately assess and record the whole grain content of foods in participant diets, with a minimum DTI of 48 g of whole grain, rather than using the weight of the whole grain food to allow for a more accurate dose assessment [56]. Products in the Australian market can claim a whole grain content from as little as 8 g per manufacturer serve or 25% whole grain and these may be consumed alongside products that are 100% whole grain, such as oats or brown rice. The recently proposed global definition for whole grains as an ingredient and as a whole grain food provides further guidance for research to assist with comparison between studies [2]. Studies also need to consider that the health outcomes from various whole grain food products may not be homogeneous, with potential differences between types of whole grains, for example, wheat versus rye versus oats versus brown rice; differing proportions of dietary fiber, and within that, soluble to insoluble fiber content; and also consideration of other components, such as beta-glucan. This has been discussed in a previous systematic review regarding cardiovascular risk factors, where whole grain oats were found to be more effective than other grains in reducing cholesterol, and brown rice was more effective in reducing triglycerides. 

A strength of this analysis was the study design, clarifying the discrepancies in previously published systematic reviews. For example, the careful inclusion of only adult RCTs, and the removal of quasi-experimental studies including only those utilizing blood measures of cytokines (not faecal measures) and those with test diets that included whole grain rather than the fiber component from whole grain sources. The collection of data from the differing population groups enabled categorization and comparison between study population types, highlighting differences between healthy and unhealthy population groups, a potential consideration for future research. 

## 5. Conclusions

With obesity rates continuing to grow in Australia and globally, coupled with the link to a higher risk of chronic disease, dietary interventions that investigate simple food changes, such as exchanging refined grain for whole grain, are of particular interest. This study further contributes to increasing current knowledge, pointing to future research considerations, particularly the need to conduct research with individual whole grain food types, discern potential differences, accurately account for the dose of whole grain, and measure compliance. 

## Figures and Tables

**Table 1:** Characteristics of studies examining whole grain consumption and inflammatory markers.

Study	Design and Duration	N (I/C)	Characteristics	(M/F)	Age (Years)	Intervention Diet	Control Diet
Ampatzoglou et al. 2016 [24]	Cr 6 weeks	33 (33/33)	Healthy	(12/21)	48.8 ± 1.1	WG > 80 g/day	RG diet; <16 g/day WG
Andersson et al. 2007 [25]	Cr 6 weeks	30 (30/30)	Overweight	(8/22)	59 ± 5	Various WGs = 112 g/day	Various RGs-111 g/day
Brownlee et al. 2010 [22]	P 16 weeks	266 (85/81/100)	Overweight	(133/133)	G1: 45.9 ± 10.1; G2: 45.7 ± 9.9; G3: 45.6 ± 1.0	G1: WG 60g/day; G2: 60 g/day 8 weeks + 120 g/day 8 weeks	Same diet as prior WG < 30 g/day
Connolly et al. 2011 [26]	Cr 16 weeks	32 (16/16)	Glucose intolerant or mild to moderate hypercholesterolamic	(12/20)	23–64	WG: 45 g WG/day as breakfast cereal	RG: 45 g/day as breakfast cereal
Giacco et al. 2013 [27]	P 12 weeks	123 (61/62)	Metabolic syndrome	N/A	40–65	WG or WW foods to replace RG	RG foods only for breads, pastas, cereals
Harris Jackson et al. 2014 [28]	P 12 weeks	50 (25/25)	Metabolic syndrome	(25/25)	35–45	187 g WG/day	RG, WG = 0 g/day
Hoevenaars et al. 2019 [29]	P 12 weeks	50 (25/25)	Overweight and obese	(19/31)	45–70	98 g WG/day	98 g RG/day
Iversen et al. 2021 [30]	P 12 weeks	242 (121/121)	Overweight and obese	(95/147)	30–70	Rye 53–60 g/day	Wheat 66 g/day
Joo et al. 2020 [31]	P 12 weeks	49 (26/23)	Metabolic syndrome	(38/11)	44.3 ± 6.1	Black rice powder 60 g/day	White rice powder 60 g/day
Katcher et al. 2008 [32]	P 12 weeks	50 (25/25)	Obese with metabolic syndrome	(25/25)	WG 45.4 ± 8; RG 46.6 ± 9.7	WG: 5, 6, 7 serves on hypocaloric diet	No WG foods in hypocaloric diet
Kazemzadeh et al. 2014 [33]	Cr 14 weeks	35 (20/15)	Overweight and obese	(0/35)	32.6 ± 6	Brown rice 150 g/day	White rice 150 g/day
Kirwan et al. 2016 [34]	Cr 8 weeks	33 (33/33)	Overweight and obese	(6/27)	39 ± 7	WG 93 ± 19 g/day	RG, WG = 0 g
Kondo et al. 2017 [35]	P 8 weeks	28 (14/14)	Type 2 Diabetes	(18/10)	40–80	Brown rice (250 cal = 182 g) to replace 10/21 meals/week	White rice (250 cal = 153 g) to replace 10/21 meals/week
Kopf et al. 2018 [36]	P 6 weeks	31 (17/14)	Overweight and obese	N/A	WG:39.2 ± 13.5 RG:27.6 ± 5.9	Whole grains 3.4 ± 0.2 serves/day	Refined grains 7.1 ± 0.7 serves/day
Li et al. 2018 [37]	Cr 8 weeks	30 (15/15)	Overweight and obese	(30/0)	36–70	20 g quinoa flour/day in form of 160 g bread roll	20 g refined flour/day in form of 160 g bread roll
Ma et al. 2013 [23]	P 30 days	199 (65/71/63)	Type 2 Diabetes & Metabolic Syndrome	(84/115)	20–65	WG1: 50 g oat germ/day	Usual diet
						WG2: 100 g oat germ/day	Usual diet
Malik et al. 2019 [38]	Cr 14 weeks	113 (55/58)	Overweight BMI > 23	(62/51)	25–65	Brown rice 182 g/day	White rice 175 g/day
Meng et al. 2018 [39]	Cr 13 weeks	11	Overweight and obese	(4/7)	50–80	Unrefined carbohydrate 19.5 g fiber/day	Refined carbohydrate 9.6 g fiber/day
Munch Roager et al. 2019 [40]	Cr 16 weeks	50 (25/25)	Overweight and obese	(18/32)	20–65	WG 157.9 ± 35 g/day	RG diet; WG 6 ± 4.8 g/day
Navarro et al. 2018 [41]	Cr 4 weeks	80	Healthy	(40/40)	18–45	Whole grain foods 55 g fiber/day	Refined grain foods 28 g fiber/day
Pavadhgul et al. 2019 [42]	Cr 8 weeks	24	Hypercholesterolamic	(12/12)	30–60	Whole grain oat porridge 70 g/day	Rice porridge 70 g/day
Pavithran et al. 2020 [43]	P 24 weeks	80 (40/40)	Type 2 diabetes	(52/28)	LGI: 54.43 ± 7.57 Control: 51.93 ± 7.43	LGI: whole wheat, red rice	Usual diet
Pourshahidi et al. 2020 [44]	Cr 12 weeks	40	Overweight and obese	(12/28)	57.68 ± 6.15	15g quinoa biscuits (60 g flour/100 g)	Control iso-energetic biscuits
Saglam et al. 2018 [45]	P 4 weeks	24 (12/12)	Type 2 Diabetes	(0/24)	40.29 ± 6.81	Whole grain bread 270 cal/ 35.32 g fiber/day	Whole wheat bread 227 cal/7.39 g fiber/day
Schutte et al. 2018 [46]	P 12 weeks	50 (25/25)	Overweight	(31/19)	WG: 61 [51–70] RG: 61 [4–69]	WG 98 g/day	RG 98 g/day
Tighe et al. 2010 [47]	P 12 weeks	136 (73/63)	Overweight	(68/68)	WG1: 51.6 ± 0.8; RG: 51.8 ± 0.8	WG1: 3 servings (70-8 0g WG bread + 30-40 g WG cereal)	Refined cereals and white bread
					WG2: 52.1 ± 0.9; RG: 51.8 ± 0.8	WG2: 1 serve of whole wheat foods + 2 serving of oats	Refined cereals and white bread
Vetrani et al. 2016 [48]	P 12 weeks	40 (21/19)	Metabolic syndrome	(16/24)	WG 57.2 ± 1.9; RG 58.4 ± 1.6	WG products plus a small portion of endosperm rye bread 40.2 ± 1.2 g fiber/day	Commercial refined grain cereal products 22.1 ± 0.9g fiber/day
Vitaglione et al. 2015 [49]	P 8 weeks	68 (36/32)	Overweight and obese	(0/68)	WG 40 ± 2; RG 37 ± 2	100% WG, 70 g/day	RG products, 60 g/day
Whittaker et al. 2015 [50]	Cr 24 weeks	22	Acute Coronary Syndrome	(13/9)	61 (47-75)	Khosoran Semolina 62 g/day Khosoran flour 140 g/day	Control Semolina 62 g/day Control Flour 140 g/day
Whittaker et al. 2017 [51]	Cr 24 weeks	21	Type 2 Diabetes	(7/14)	64.4 ± 10.9 w	Khosoran Semolina 62 g/day Khosoran flour 140 g/day	Control Semolina 62 g/day Control Flour 140 g/day
Zamaratskaia et al. 2020 [52]	Cr 24 weeks	17	Prostate cancer	(17/0)	73.5 ± 4.6	WG foods 485 g/day	RG foods 485 g/day

Abbreviations: Crossover (Cr); Parallel (P); Number of participants (*n*); Intervention (I); Control (C); Male (M); Female (F); Whole Grain (WG); Whole Wheat (WW); Refined Grain (RG); Group (G).

**Table 2:** Effect of whole grain consumption on inflammatory markers in healthy individuals between the intervention and control diet.

**Study**	**N (I/C)**	**CRP Baseline**	**CRP Endpoint**	***p*-Value**
Ampatzoglou et al. 2016 [24]	I (*n* = 33)	2.2 (0.5) ng/L	1.6 (0.4) ng/L	0.099
	C (*n* = 33)	1.7 (0.3) ng/L	1.8 (0.3) ng/L	
Navarro et al. 2019 [41]	I (*n* = 40)	1.5 ± 2.7 mg/L	n.d	0.19
	C (*n* = 40)	1.5 ± 2.7 mg/L	n.d	
**Study**	**N (I/C)**	**IL-6 Baseline**	**IL-6 Endpoint**	***p*-Value**
Ampatzoglou et al. 2016 [24]	I (*n* = 33)	1.2 (0.2) ng/L	1.6 (0.1) ng/L	0.702
	C (*n* = 33)	1.3 (0.2) ng/L	1.4 (0.2) ng/L	
**Study**	**N (I/C)**	**TNF Baseline**	**TNF Endpoint**	***p*-Value**
Ampatzoglou et al. 2016 [24]	I (*n* = 33)	10.8 (0.4) ng/L	10.8 (0.6) ng/L	0.381
	C (*n* = 33)	10.5 (0.5) ng/L	10.7 (0.5) ng/L	

**Abbreviations:** Number of participants (N); Intervention (I); Control (C); C-Reactive Protein (CRP); Interlukin-6 (IL-6); Tumor Necrosis Factor (TNF); *p*-value between groups unless stated; *p*-value < 0.05; baseline and endpoint data presented as mean ± S.D, mean (range) or mean (SE) as per raw data, where S.D is standard deviation and SE = standard error.

**Table 3:** Effect of whole grain consumption on inflammatory markers in overweight and obese individuals.

**Study**	**N (I/C)**	**CRP Baseline**	**CRP Endpoint**	***p*-Value**
Andersson et al. 2007 [25]	I (*n* = 30)	2.03 ± 1.62 mg/L	2.38 ± 2.29 mg/L	0.55
	C (*n* = 30)	2.86 ± 2.96 mg/L	2.34 ± 1.57 mg/L	
Brownlee et al. 2010 [22]	I1 (*n* = 85)	2.4 ± 9.9 mg/L	3.1 ± 4.3 mg/L	>0.05
	C (*n* = 100)	2.4 ± 2.3 mg/L	2.9 ± 3.5 mg/L	
Brownlee et al. 2010 [22]	I2 (*n* = 81)	3.2 ± 4.6 mg/L	3.2 ± 5.9 mg/L	>0.05
	C (*n* = 100)	2.4 ± 2.3 mg/L	2.9 ± 3.5 mg/L	
Hoevenaars et al. 2019 [29]	I (*n* = 20)	5.29 ± 8.14 μg/mL	2.16 ± 1.82 μg/mL	0.03 **
	C (*n* = 20)	2.58 ± 2.70 μg/mL	5.24 ± 14.1 μg/mL	
Iversen et al. 2021 [30]	I (*n* = 121)	1.45 (1.21; 1.73) mg/L	1.12 (0.93; 1.36) mg/L	0.001 **
	C (*n* = 121)	1.44 (1.19; 1.74) mg/L	1.58 (1.29; 1.92) mg/L	
Katcher et al. 2008 [32]	I (*n* = 121)	1.45 (1.21; 1.73)	1.12 (0.93; 1.36) mg/L	0.001 **
	C (*n* = 121)	1.44 (1.19; 1.74)	1.58 (1.29; 1.92) mg/L	
Kazemzadeh et al. 2014 [33]	I (*n* = 20)	G1: 2.0 ± 1.3 mg/L G2: 1.5 ± 1.2 mg/L	G1: 1.9 ± 1.9 mg/L G2: 0.9 ± 1.1 mg/L	0.012 **
	C (*n* = 15)	G1: 2.0 ± 1.3 mg/L G2: 1.5 ± 1.2 mg/L	G1: 1.9 ± 1.9 mg/L G2: 0.9 ± 1.1 mg/L	
Kirwan et al. 2016 [34]	I (*n* = 33)	3.7 ± 3.3 mg/L	0.8 (−1.1, 2.6) mg/L	0.06
	C (*n* = 33)	5.9 ± 7.1 mg/L	−2.3 (−4.8, 0.1) mg/L	
Kopf et al. 2018 [36]	I (*n* = 17)	0.8 ± 0.6 mg/mL	0.8 ± 0.4 mg/mL	0.89
	C (*n* = 14)	0.6 ± 0.4 mg/mL	0.7 ± 0.5 mg/mL	
Li et al. 2018 [37]	I (*n* = 28)	3.7 ± 3.3 mg/L	3.7 ± 3.3 mg/L	0.197
	C (*n* = 28)	3.7 ± 3.3 mg/L	3.7 ± 3.3 mg/L	
Malik et al. 2019 [38]	I (*n* = 55)	4.1 ± 2.8 mg/L	0.03 ± 2.12 mg/L	0.04 **
	C (*n* = 58)	4.1 ± 2.8 mg/L	0.63 ± 2.35 mg/L	
Meng et al. 2019 [39]	I (*n* = 11)	n.d	2.1 (0.7–4.7) mg/L	0.84
	C (*n* = 11)	n.d	2.0 (0.6–4.6) mg/L	
Munch Roager et al. 2019 [40]	I (*n* = 25)	6.3 ± 14.0 mg/L	4.2 ± 6.8 mg/L	0.003 **
	C (*n* = 25)	3.1 ± 2.6 mg/L	5.0 ± 5.8 mg/L	
Pourshahidi et al. 2020 [44]	I (*n* = 20)	156 ± 195 μg/dL	142 ± 115 μg/dL	0.265
	C (*n* = 20)	156 ± 195 μg/dL	171 ± 254 μg/dL	
Schutte et al. 2018 [46]	I (*n* = 25)	5294 ± 8140 ng/mL	2162 ± 7260 ng/mL	0.064
	C (*n* = 25)	2575 ± 2702 ng/mL	2555 ± 1658 ng/mL	
Tighe et al. 2010 [47]	I1 (*n* = 85)	3.3 (0.5, 2.3) mg/L	0.9 (0.5, 1.9) mg/L	0.349
	C (*n* = 100)	1.4 (0.7, 2.7) mg/L	1.1 (0.6, 3.0) mg/L	
Tighe et al. 2010 [47]	I2 (*n* = 81)	1.0 (0.4, 1.6) mg/L	1.0 (0.6, 2.3) mg/L	0.349
	C (*n* = 100)	1.4 (0.7, 2.7) mg/L	1.1 (0.6, 3.0) mg/L	
**Study**	**N (I/C)**	**IL-6 Baseline**	**IL-6 Endpoint**	***p*-Value**
Andersson et al. 2007	I (*n* = 30)	14.8 ± 32.2 mg/L	15.2 ± 33.2 mg/L	0.79
[25]	C (*n* = 30)	15.9 ± 32.4 mg/L	15.8 ± 30.9 mg/L	
Hoevenaars et al. 2019	I (*n* = 20)	1.17 ± 1.26 pg/mL	1.13 ± 0.89 pg/mL	0.73
[29]	C (*n* = 20)	1.09 ± 0.81 pg/mL	1.46 ± 1.58 pg/mL	
Katcher et al. 2008 [32]	I (*n* = 121)	3.2 ± 6.3 pg/mL^6	2.3 ± 3.6 pg/mL^6	Group 0.94 ^
	C (*n* = 121)	2.2 ± 1.3 pg/mL^6	2.1 ± 0.4 pg/mL^6	Time 0.57
Kopf et al. 2018 [36]	I (*n* = 17)	4.4 ± 1.9 mg/mL	5.2 ± 1.3 mg/mL	0.89
	C (*n* = 14)	2.9 ± 1.5 mg/mL	3.2 ± 1.7 mg/mL	
Meng et al. 2019 [39]	I (*n* = 11)	n.d	0.6 (0.4–0.8) pg/L	0.77
	C (*n* = 11)	n.d	0.6 (0.4–0.8) pg/L	
Munch Roager et al. 2019 [40]	I (*n* = 20)	1.6 ± 1.2 mg/L	1.4 ± 1.1 mg/L	0.009 **
	C (*n* = 15)	1.2 ± 0.7 mg/L	2.0 ± 2.0 mg/L	
Tighe et al. 2010 [47]	I1 (*n* = 85)	1.3 (0.8, 2.3) pg/L	1.4 (1.0, 2.4) pg/L	>0.05
	C (*n* = 100)	1.1 (0.8, 1.7) pg/L	1.1 (0.8, 1.6) pg/L	
Tighe et al. 2010 [47]	I2 (*n* = 81)	1.2 (0.9, 1.9) pg/L	0.9 (0.5, 1.9) pg/L	>0.05
	C (*n* = 100)	1.1 (0.8, 1.7) pg/L	1.1 (0.8, 1.6) pg/L	
Vitaglione et al. 2015 [49]	I (*n* = 36)	57.5 ± 7.5 pg/mL	46.9 ± 4.0 pg/mL	0.06
	C (*n* = 32)	65.5 ± 11.4 pg/mL	60.2 ± 7.2 pg/mL	
**Study**	**N (I/C)**	**TNF Baseline**	**TNF Endpoint**	***p*-Value**
Hoevenaars et al. 2019	I (*n* = 20)	3.07 ± 1.85 pg/mL	2.90 ± 1.89 pg/mL	0.26
[29]	C (*n* = 20)	2.26 ± 1.43 pg/mL	2.29 ± 1.38 pg/mL	
Katcher et al. 2008 [32]	I (*n* = 121)	1.2 ± 0.3 pg/mL^6	1.1 ± 0.3 pg/mL^6	Group 0.04 **^
	C (*n* = 121)	1.3 ± 0.4 pg/mL^6	1.2 ± 0.2 pg/mL^6	Time 0.80
Kopf et al. 2018 [36]	I (*n* = 17)	26.7 ± 4.17 pg/mL	21.4 ± 2.9 pg/mL	0.11
	C (*n* = 14)	23.8 ± 5.9 pg/mL	23.4 ± 6.6 pg/mL	
Munch Roager et al. 2019 [40]	I (*n* = 20)	1.7 ± 0.8 pg/mL	1.7 ± 0.08 pg/mL	0.87
	C (*n* = 15)	1.7 ± 0.9 pg/mL	1.7 ± 0.9 pg/mL	
Vitaglione et al. 2015 [49]	I (*n* = 36)	341.9 ± 25.5 pg/mL	26.8 ± 3.2 pg/mL	0.04 **
	C (*n* = 32)	321.9 ± 52.1 pg/mL	329.8 ± 5.06 pg/mL	

Abbreviations: Number of participants (N); Intervention (I); Control (C); C-Reactive Protein (CRP); Interlukin-6 (IL-6); Tumor Necrosis Factor (TNF); *p*-value between group unless stated; *p*-value < 0.05 (**); baseline and endpoint data presented as mean ± SD, mean (range) or mean (SE) as per raw data, where SD is standard deviation and SE = standard error; ^ *p*-value Group vs. Time.

**Table 4:** Effect of whole grain consumption on inflammatory markers in individuals with pre-existing conditions.

**Study**	**N (I/C)**	**CRP Baseline**	**CRP Endpoint**	***p*-Value**
Connolly et al. 2011 [26]	I (*n* = 16)	1.69 ± 0.35 mg/L	2.45 ± 0.92mg/L	0.934
	C (*n* = 16)	1.8 ± 0.47 mg/L	2.36 ± 0.49 mg/L	
Giacco et al. 2013 [27]	I (*n* = 61)	1.95 (0.74; 4.12) mg/dl	1.36 (0.62; 3.34) mg/dl	0.16
	C (*n* = 62)	1.95 (0.96; 2.56) mg/dl	1.74 (1.04; 2.95) mg/dl	
Harris Jackson et al. 2014 [28]	I (*n* = 17)	3.0 (2.0, 4.6) mg/L	2.4 ± 0.5 mg/L	>0.05
	C (*n* = 25)	2.1 (1.4, 3.1) mg/L	1.5 ± 0.4 mg/L	
Joo et al. 2020 [31]	I (*n* = 26)	0.205 (0.183) mg/dL	0.101 (0.028) mg/dL	0.03 **
	C (*n* = 23)	0.137 (0.165) mg/dL	0.154 (0.025) mg/dL	
Kondo et al. 2017 [35]	I (*n* = 14)	0.09 ± 0.12 μg/L	0.05 ± 0.05 μg/L	0.063
	C (*n* = 14)	0.04 ± 0.03 μg/L	0.05 ± 0.06 μg/L	
Ma et al. 2013 [23]	I1 (*n* = 65)	3.65 (2.45) mg/L	3.13 (2.61) mg/L	>0.05
	C (*n* = 63)	3.76 (1.99) mg/L	3.81 (2.21) mg/L	
Ma et al. 2013 [23]	I2 (*n* = 71)	3.46 (2.55) mg/L	2.26 (2.12) mg/L	<0.05 **
	C (*n* = 63)	3.76 (1.99) mg/L	3.81 (2.21) mg/L	
Pavadhgul et al. 2019 [42]	I (*n* = 24)	2.7 ± 2.1 mg/L	2.2 ± 2.1 mg/L	<0.05 **
	C (*n* = 24)	2.7 ± 2.1 mg/L	2.9 ± 2.9 mg/L	
Pavithran et al. 2020 [43]	I (*n* = 40)	3.38 ± 3.83 mg/L	1.46 ± 1.04 mg/L	0.026 **
	C (*n* = 40)	2.79 ± 4.20 mg/L	3.16 ± 4.61 mg/L	
Saglam et al. 2019 [45]	I (*n* = 12)	n.d	n.d	>0.05
	C (*n* = 12)	n.d	n.d	
Vetrani et al. 2016 [48]	I (*n* = 21)	2.52 ± 0.5 mg/dL	2.44 ± 0.5 mg/dL	0.693
	C (*n* = 19)	2.27 ± 0.4 mg/dL	2.39 ± 0.4 mg/dL	
**Study**	**N (I/C)**	**IL-6 Baseline**	**IL-6 Endpoint**	***p*-Value**
Connolly et al. 2011 [26]	I (*n* = 16)	4.13 ± 1.47 pg/mL	5.88 ± 1.78 pg/mL	0.925
	C (*n* = 16)	4.09 ± 1.71 pg/mL	7.16 ± 3.46 pg/mL	
Giacco et al. 2013 [27]	I (*n* = 61)	1.42 (1.01; 2.32) pg/mL	1.54 (1.12; 2.23) pg/mL	0.52
	C (*n* = 62)	1.41 (0.84; 2.21) pg/mL	1.43 (1.07; 2.11) pg/mL	
Harris Jackson et al. 2014 [28]	I (*n* = 23)	1.8 (1.5, 2.2) pg/mL	2.1 ± 0.2 pg/mL	>0.05
	C (*n* = 23)	1.7 (1.4, 2.0) pg/mL	1.8 ± 0.2 pg/mL	
Pavadhgul et al. 2019 [42]	I (*n* = 24)	150 ± 57.9 pg/L	123 ± 44.5 pg/L	<0.01 **
	C (*n* = 24)	150 ± 57.9 pg/L	145 ± 54.0 pg/L	
Vetrani et al. 2016 [48]	I (*n* = 21)	1.84 ± 0.2 pg/mL	2.23 ± 0.3 pg/mL	0.161
	C (*n* = 19)	1.69 ± 0.3 pg/mL	1.7 ± 0.3 pg/mL	
Whittaker et al. 2015 [50]	I (*n* = 22)	2.26 (1.50–3.03) pg/mL	1.53 (1.16–1.90) pg/mL	0.698
	C (*n* = 22)	3.16 (1.51–4.81) pg/mL	3.30 (1.24–6.37) pg/mL	
Whittaker et al. 2017 [51]	I (*n* = 21)	2.76 ± 2.01 pg/mL	2.16 ± 1.21 pg/mL	0.9
	C (*n* = 21)	2.15 ± 1.57 pg/mL	1.70 ± 1.24 pg/mL	
**Study**	**N (I/C)**	**TNF Baseline**	**TNF Endpoint**	***p*-Value**
Connolly et al. 2011 [26]	I (*n* = 16)	20.2 ± 4.0 pg/mL	36.5 ± 15.7 pg/mL	0.519
	C (*n* = 16)	46.3 ± 26.0 pg/mL	42.2 ± 14.8 pg/mL	
Giacco et al. 2013 [27]	I (*n* = 61)	0.73 (0.50; 0.96) pg/mL	0.68 (0.50; 0.94) pg/mL	0.84
	C (*n* = 62)	0.62 (0.43; 1.05) pg/mL	0.63 (0.41; 0.90) pg/mL	
Harris Jackson et al. 2014 [28]	I (*n* = 24)	1.2 (1.0, 1.3) pg/mL	1.2 ± 0.1 pg/mL	<0.05 **
	C (*n* = 24)	1.4 (1.2, 1.7) pg/mL	1.3 ± 0.1^5 pg/mL	
Pavadhgul et al. 2019 [42]	I (*n* = 24)	49.5 ± 26.4 pg/L	39.83 ± 15.9 pg/L	<0.01 **
	C (*n* = 24)	49.5 ± 26.4 pg/L	47.4 ± 24.1 pg/L	
Vetrani et al. 2016 [48]	I (*n* = 21)	1.71 ± 0.6 pg/mL	1.50 ± 0.6 pg/mL	0.232
	C (*n* = 19)	1.07 ± 0.4μg/mL	1.31 ± 0.5 pg/mL	
Whittaker et al. 2015 [50]	I (*n* = 22)	4.54 ± 3.32 pg/mL	3.9 (1.4–6.4) pg/mL	0.798
	C (*n* = 22)	6.5 (2.9–9.9) pg/mL	4.6 (0.9–8.2) pg/mL	
Whittaker et al. 2017 [51]	I (*n* = 21)	4.54 ± 3.32 pg/mL	4.74 ± 3.09 pg/mL	0.04 **
	C (*n* = 21)	4.36 ± 4.09 pg/mL	4.84 ± 4.07 pg/mL	

Abbreviations: Number of participants (N); Intervention (I); Control (C); C-Reactive Protein (CRP); Interlukin-6 (IL-6); Tumor Necrosis Factor (TNF); *p*-value between group unless stated; *p*-value < 0.05 (**); baseline and endpoint data presented as mean ± SD, mean (range) or mean (SE) as per raw data, where SD is standard deviation and SE = standard error.

**Table 5:** Effect of whole grain consumption on inflammatory markers in individuals with other conditions.

**Study**	**N (I/C)**	**CRP Baseline**	**CRP Endpoint**	***p*-Value**
Zamaratskaia et al. 2020	I (*n* = 17)	n.d	n.d	>0.05
[52]	C (*n* = 17)			
**Study**	**N (I/C)**	**IL-6 Baseline**	**IL-6 Endpoint**	***p*-Value**
Zamaratskaia et al. 2020	I (*n* = 17)	6.3 (5.3–7.5) pg/mL	n.d	>0.05
[52]	C (*n* = 17)	5.8 (4.8–6.9) pg/mL		

Abbreviations: Number of participants (N); Intervention (I); Control (C); C-Reactive Protein (CRP); Interlukin-6 (IL-6); *p*-value between group unless stated; *p*-value <0.05; baseline and endpoint data presented as mean ± SD, mean (range) or mean (SE) as per raw data, where SD is standard deviation and SE = standard error.

## Supplementary Materials

The following are available online at https://www.mdpi.com/article/10.3390/nu14020374/s1, Table S1: PICO Framework, Table S2: Search Terms, Figures S1 and S2: Results of Risk of Bias Assessment.
