# Supplementary material for: Whole Grain Consumption and Inflammatory Markers: A Systematic Literature Review of Randomized Control Trials
Source: Nutrients. 2022 Jan 16;14(2):374. doi: 10.3390/nu14020374 (PMC8778110; doi:10.3390/nu14020374)
Supplement: Supplementary file 1 [file nutrients-14-00374-s001.zip › nutrients-1531144-supplementary.pdf]

nutrients

Supplementary Materials

Table S1. PICO framework: 'Is there an effect of wholegrain consumption on measures of inflammation for adults?

**PICO Categories**	**Included**	**Excluded**
Participants	Adults (aged ≥18 years)	Children (aged < 18 years)
Interventions	Consumption of a whole or pseudo grain<br>Cereal Grains; Wheat; including Spelt, Emmer, Einkorn, Khorasan or Kamut, Durum, and Faro; Oats, Corn/Maize, Rice, Teff, Canary Seed, Job's Tears, Barley, Sorghum, Rye, Millet and Triticale, and Pseudo-Cereal Grains; Amaranth, Buckwheat, Quinoa and Wild Rice;	Does not meet the criteria for whole or pseudo grain
Control / Comparator	Refined grain, alternate food or placebo	No comparator or control in study design
Outcomes	Change in ≥1 serum inflammatory markers;<br>interleukin-6, (IL-6), C-reactive protein, (CRP), tumour necrosis factor, (TNF).	No reporting on inflammatory marker change

Figure S1. Inclusion and Exclusion criteria of a systematic review and meta-analysis of randomized control trials that compared whole pseudo-grain consumption in humans.

Table S2: Search terms (MEDLINE)

---

1 Whole Grains/
2 whole grain.mp.
3 whole wheat.mp.
4 corn.mp.
5 rice.mp.
6 oat*.mp.
7 maize*.mp.
8 barley.mp.
9 sorghum*.mp.
10 rye*.mp.
11 millet.mp.
12 triticale.mp.
13 amaranth.mp.
14 quinoa.mp.
15 wild rice*.mp.
16 spelt.mp.
17 emmer.mp.
18 einkorn.mp.
19 khorasan.mp.
20 kamut.mp.
21 durum.mp.
22 fari.mp.
23 teff.mp.
24 canary seed.mp.
25 jobs tears.mp.
26 wholegrain.mp.
27 1 or 2 or 3 or 4 or 5 or 6 or 7 or 8 or 9 or 10 or 11 or 12 or 13 or 14 or 15 or 16 or 17 or 18 or 19 or 20 or 21 or 22 or 23 or 24 or 25 or 26
28 Inflammation/
29 inflammation.mp.
30 C-Reactive Protein/
31 Tumo?r Necrosis Factor.mp.
32 Interleukin-6/
33 interlukin-6.mp.
34 CRP.tw.
35 TNF.tw.
36 IL.tw.
37 IL-6.tw.
38 28 or 29 or 30 or 31 or 32 or 33 or 34 or 35 or 36 or 37
39 27 and 38
40 limit 39 to humans
41 limit 40 to ("all adult (19 plus years)" or "young adult (19 to 24 years)" or "adult (19 to 44 years)" or "young adult and adult (19-24 and 19-44)" or "middle age (45 to 64 years)" or "middle aged (45 plus years)" or "all aged (65 and over)" or "aged (80 and over)")
42 limit 41 to randomized controlled trial
43 cereal.mp.
44 cereal*.mp.
45 grain*.mp.
46 oat*.mp.
47 wheat.mp.
48 bran.mp.
49 barley.mp.

50 whole grain.mp.
51 refined grain.mp.
52 triticale.mp.
53 43 or 44 or 45 or 46 or 47 or 48 or 49 or 50 or 51 or 52
54 cytokines.mp.
55 interlukin.mp.
56 inflammation.mp.
57 inflammation mediators.mp.
58 IL.mp.
59 C-Reactive protein.mp.
60 CRP.mp.
61 inflammatory.mp.
62 tumo?r necrosis factor.mp.
63 TNF.mp.
64 interlukins.mp.
65 54 or 55 or 56 or 57 or 58 or 59 or 60 or 61 or 62 or 63 or 64
66 53 and 65
67 limit 66 to humans
68 limit 67 to ("all adult (19 plus years)" or "young adult (19 to 24 years)" or "adult (19 to 44 years)" or "young adult and adult (19-24 and 19-44)" or "middle age (45 to 64 years)" or "middle aged (45 plus years)" or "all aged (65 and over)" or "aged (80 and over)")
69 limit 68 to randomized controlled trial
70 limit 69 to english language

Figure S1: Cochrane Rob2 for Crossover Studies raw data

Study	D1	DS	D2	D3	D4	D5	Overall
Ampatzoglou et al. 2016 [24]	!	+	+	+	+	+	!
Andersson et al. 2007 [25]	+	+	+	+	!	+	!
Connolly et al. 2011 [26]	+	+	+	+	+	!	!
Kazemzadeh et al. 2014 [33]	+	+	+	+	+	+	+
Kirwan et al. 2016 [34]	+	+	+	+	+	+	+
Li et al. 2018 [37]	+	+	+	+	+	+	+
Malik et al. 2019 [38]	+	+	+	+	+	+	+
Meng et al 2018 [39]	+	+	+	+	+	+	+
Munch Roager et al. 2019 [40]	+	+	+	+	+	+	+
Navarro et al. 2019 [41]	+	+	+	!	+	+	!
Pavadhgul et al. 2019 [42]	+	+	!	+	+	+	!
Pourshahidi et al. 2020 [44]	+	+	+	+	+	+	+
Whittaker et al. 2015 [50]	+	+	+	+	+	+	+
Whittaker et al. 2017 [51]	+	+	+	+	+	+	+
Zamaratskaia et al. 2020 [52]	+	+	+	+	+	+	+

Legend:

+	Low risk
!	Some concerns
-	High risk
DS	Bias arising from period and carryover effects
D1	Randomisation process
D2	Deviations from the intended interventions
D3	Missing outcome data
D4	Measurement of the outcome
D5	Selection of the reported result

Figure S2: Cochrane Rob2 for Parallel Studies raw data

Study	D1	D2	D3	D4	D5	Overall
Brownlee et al. 2010 [22]	+	-	+	+	+	!
Giacco et al. 2013 [27]	+	+	+	+	+	+
Harris Jackson et al. 2014 [28]	!	+	!	+	+	!
Hoevenaars et al. 2019 [29]	+	+	+	+	+	+
Iversen et al. 2021 [30]	!	+	+	+	+	!
Joo et al. 2020 [31]	!	!	+	+	!	!
Katcher et al. 2008 [32]	+	+	+	+	+	+
Kondo et al. 2017 [35]	+	+	+	+	+	+
Kopf et al. 2018 [36]	+	+	+	+	+	+
Ma et al. 2013 [23]	+	+	+	+	+	+
Pavithran et al. 2020 [43]	!	+	+	+	+	!
Saglam et al. 2018 [45]	+	+	!	+	+	!
Schutte et al. 2018 [46]	+	+	+	+	+	+
Tighe et al. 2010 [47]	+	+	+	!	+	!
Vetrani et al. 2016 [48]	+	+	+	+	+	+
Vitaglione et al. 2015 [49]	+	+	+	+	+	+

Legend:

+	Low risk
!	Some concerns
-	High risk
D1	Randomisation process
D2	Deviations from the intended interventions
D3	Missing outcome data
D4	Measurement of the outcome
D5	Selection of the reported result
